# Selection of reference genes for RT-qPCR analysis in a predatory biological control agent, *Coleomegilla maculata* (Coleoptera: Coccinellidae)

**DOI:** 10.1038/srep18201

**Published:** 2015-12-10

**Authors:** Chunxiao Yang, Huipeng Pan, Jeffrey Edward Noland, Deyong Zhang, Zhanhong Zhang, Yong Liu, Xuguo Zhou

**Affiliations:** 1 Hunan Academy of Agricultural Sciences, Institute of Plant Protection, Hunan, China; 2 Department of Entomology, University of Kentucky, Lexington, KY, USA; 3 Hunan Vegetable Institute, Hunan Academy of Agricultural Sciences, Hunan, China

## Abstract

Reverse transcriptase-quantitative polymerase chain reaction (RT-qPCR) is a reliable technique for quantifying gene expression across various biological processes, of which requires a set of suited reference genes to normalize the expression data. *Coleomegilla maculata* (Coleoptera: Coccinellidae), is one of the most extensively used biological control agents in the field to manage arthropod pest species. In this study, expression profiles of 16 housekeeping genes selected from *C. maculata* were cloned and investigated. The performance of these candidates as endogenous controls under specific experimental conditions was evaluated by dedicated algorithms, including *geNorm*, *Normfinder*, *BestKeeper*, and Δ*Ct* method. In addition, *RefFinder*, a comprehensive platform integrating all the above-mentioned algorithms, ranked the overall stability of these candidate genes. As a result, various sets of suitable reference genes were recommended specifically for experiments involving different tissues, developmental stages, sex, and *C. maculate* larvae treated with dietary double stranded RNA. This study represents the critical first step to establish a standardized RT-qPCR protocol for the functional genomics research in a ladybeetle *C. maculate*. Furthermore, it lays the foundation for conducting ecological risk assessment of RNAi-based gene silencing biotechnologies on non-target organisms; in this case, a key predatory biological control agent.

RNA interference (RNAi) is a sequence-specific post-transcriptional gene silencing process elicited by double stranded RNA (dsRNA) that occurs widely among plants, animals, and microorganisms^1^. In recent years, the development of RNAi-based transgenic technology, especially *in planta* RNAi, has seen a rapid growth and offers a novel approach for the sustainable management of insect pests^2^,^3^,^4^,^5^,^6^,^7^,^8^,^9^,^10^. Transgenic crops expressing long dsRNAs to control Coleopteran pests, e.g., western corn rootworm, *Diabrotica virgifera virgifera* LeConte, is at the forefront of the research and development efforts^4^. This trait is expected to be the first RNAi-based insect control product to be commercialized, potentially by the end of this decade^11^,^12^.

One of the major ecological concerns regarding the RNAi-based gene silencing biotechnologies is their potential adverse impacts on non-target organisms (NTOs)^13^,^14^,^15^,^16^,^17^. The surrogate NTOs, including pollinators, soil decomposers, and biological control agents, represent diverse ecological functions. Deleterious effects on NTOs tend to lead to adverse impacts on environment and compromised crop performance.

The pink spotted ladybeetle, *Coleomegilla maculata* (Coleoptera: Coccinellidae), is one of the most common and widely applied predatory natural enemy against arthropod pests, including aphids, thrips, mites, and lepidopteran and coleopteran larvae and eggs. In addition, *C. maculata* can feed on plant tissues as well, such as pollen and nectar in maize and other cropping systems^18^,^19^,^20^,^21^,^22^. As a surrogate NTO, *C. maculata* has been used extensively to evaluate the potential non-target risks of *Bacillus thuringiensis* (Bt) transgenic crops^22^,^23^,^24^,^25^,^26^,^27^,^28^,^29^,^30^,^31^. Consequently, it is germane to adopt *C. maculate* as a surrogate species to assess the risks associated with RNAi-based insecticides and transgenic crops. Given the nature of RNAi mechanisms, non-target effects will likely come down to the unexpected modulation of gene expressions in non-target organisms^32^.

Reverse transcriptase-quantitative polymerase chain reaction (RT-qPCR), a premier molecular biology tool specifically for quantification of gene expression in real-time, is a logic choice to evaluate the potential non-target impacts of this paradigm-shifting biotechnology. Although RT-qPCR is one of the most efficient, reliable, and reproducible techniques to quantify gene expression, multiple factors, including the quality and integrity of RNA samples, efficiency of cDNA synthesis, and PCR efficiency, can significantly influence the normalization processes^33^,^34^,^35^,^36^,^37^,^38^,^39^,^40^. Bustin and colleagues^38^ carried out a mega-analysis of over 1,700 peer-reviewed journal articles published in two time periods (2009–2011 and 2012–2013, respectively) whose authors use RT-qPCR analysis in their research. The surveys assessed the quality of these publication based on four key parameters, including RNA quality, reverse transcription conditions, PCR assay details and data analysis methodology. Although more researchers start to embrace and to adopt the Minimum Information for Publication of Quantitative Real-Time PCR Experiments (MIQE) guidelines, authors concluded that “the integrity of the scientific literature that depends upon qPCR data is severely challenged.” Similarly, authors found that normalization procedures in these surveyed papers were inadequate and insufficient^36^. The normalization bias caused by a single, non-validated reference gene has been shown to lead to unreliable results and questionable conclusions, especially with tissue samples^33^,^40^. To counter this bias, using two to five validated stably expressed reference genes is the most appropriate approach to normalize RT-qPCR data^41^.

Despite the demonstrated necessity for systematic selection and validation of reference genes in RT-qPCR studies^42^, insufficient normalization, especially, relying on non-validated (single) reference genes is still a common practice^38^,^39^. This is of particular concern as the risks associated with RNAi-based gene silencing biotechnologies on NTOs could be subtle changes in gene expression. Without sufficient selection and validation, unreliable gene expression results can lead to erroneous risk assessments and risk decisions.

The overall goal of this study is to select a suite of reference genes with stable expression under specific experimental conditions in *C. maculata*. To archive this goal, 16 housekeeping genes extracted from NCBI as well as a *C. maculata* transcriptome were chosen as the candidate reference genes^43^, including *β-actin* (*Actin*), *elongation factor 1 α* (*EF1A*), *glyceralde hyde-3-phosphate dehydrogenase* (*GAPDH*), *arginine kinase* (*ArgK*), *vacuolar-type H*^+^-*ATPase subunit A* (*V-ATPase*), *16S ribosomal RNA* (*16S*), *12S ribosomal RNA* (*12S*), *28S ribosomal RNA* (*28S*), *18S ribosomal RNA* (*18S*), *ribosomal protein S24* (*RPS24*), *heat shock protein 70* (*HSP70*), *heat shock protein 90* (*HSP90*), *a-tubulin* (*Tubulin*), *NADH dehydrogenase subunit 2* (*NADH*), *ribosomal protein S18* (*RPS18*), and *ribosomal protein L4* (*RPL4*). The stability of these candidate genes was investigated under one abiotic (dietary RNAi) and three biotic (developmental stage, tissue type, and sex) conditions. As a result, different sets of reference genes were recommended accordingly based on each experimental condition.

## Results

### Performance of RT-qPCR primers

**Figure 1:**
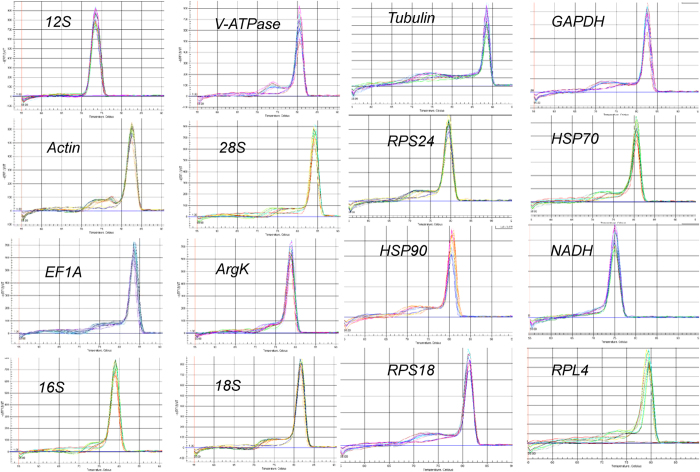
Melting curves of the 16 candidate reference genes.

All gene candidates tested were visualized as a single amplicon of expected size on a 2.0% agarose gel (Figure S1). Furthermore, gene-specific amplification was confirmed by a single peak in the melting-curve analysis (Fig. 1). The linear regression equation, correlation coefficient, and PCR efficiency for each standard curve are shown in Table 1. Additionally, the standard curve of each gene is shown in Figure S2.

### C_t_ values of candidate reference genes

**Figure 2:**
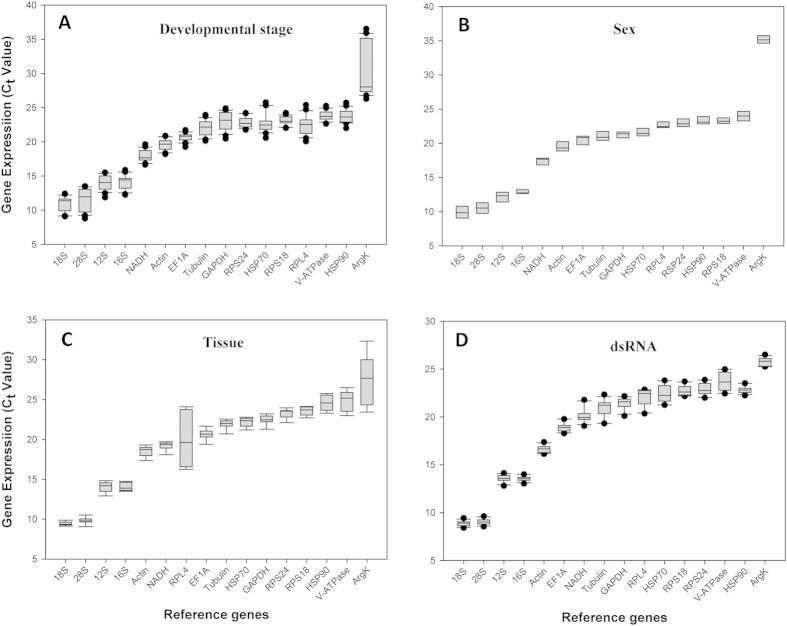
Expression profiles of the 16 candidate reference genes in all four experiments.

The *C*_*t*_ values of these 16 candidate reference genes under the four experimental conditions ranged between 9 and 35. The average *C*_*t*_ value of the four ribosomal genes, including *18S*, *28S*, *12S*, and *16S*, was under 15 cycles. *Actin* and *NADH* showed an averaged *C*_*t*_ value of less than 20 cycles. The averaged *C*_*t*_ values of *EF1A*, *GAPDH*, *Tubulin*, *RPS24*, *HSP70*, *HSP90*, *RPS18*, *RPL4*, and *V-ATPase* were between 20 and 25 cycles. *18S* and *ArgK* were the most and the least expressed reference gene, respectively (Fig. 2).

### Stability of candidate reference genes under specific experimental conditions

Developmental stages included eggs, all four larval instars (collected at the first day of each instar), pupae, adult females and males. Tissues, including head, gut, and carcass, were dissected from *C. maculata* larvae of various instars. For the sex, gene expression profiles were, respectively, investigated in adult females and males. For dietary RNAi study, four dietary treatments were included; artificial diets containing dsRNAs from dsDVV, dsCM, dsGUS, and H_2_O (vehicle control). The average expression stability value (M-value) is used by *geNorm* to determine the best set of reference genes. Recommended M values for *geNorm* are M < 0.5 for homogeneous samples and M < 1 for heterogenous samples. Here, the lower the M-value coefficient, the higher the stability ranking. Developmental stage analyses showed *RPS24* and *RPS18* were co-ranked as the most stable genes. Tissue-specific experiments indicated that *Tubulin* and *GAPDH* were the most stable genes. Sex results showed that *HSP70* and *RPS24* were co-ranked as the most stable genes. Dietary RNAi treatment revealed that *12S* and *18S* were the most stable genes. Table 2 shows the overall ranking of these reference gene candidates from the most-to-least stable ones under each experimental condition.

A low stability value (SV) suggests a more stable gene by *NormFinder*. For the developmental stage experiment, *V-ATPase* was the most stable gene. Tissue-specific experiments indicated that *12S* was the most stable gene. Sex results showed that *16S* was the most stable gene. The *18S* gene was considered the most stable for the dietary RNAi treatment experiment. The overall order based on *NormFinder* from the most-to-least stable reference genes is shown in Table 2.

The stability of a gene is inversely proportional to the standard deviation (SD) value as computed by *BestKeeper* program. Those with SD > 1 are excluded. *EF1A* was determined to be the most stable gene for the developmental stage experiment, compared to the tissue experiment where *16S* was considered to be the most stable. *GAPDH* was the most stable gene for both sexes. *18S* was shown to be the most stable gene for RNAi experiments. The overall order based on *BestKeeper* from the most-to-least stable reference genes are also found in Table 2.

The Δ*C*_*t*_ method depends on a concept similar to that of *geNorm*, it also relies on relative pair-wise comparisons. Using raw *C*_*t*_values, the average SD of each gene set is inversely proportional to its stability. Here, *V-ATPase* was the most stable gene for the developmental stage experiment, while to the tissue-specific experiments, where was shown *18S* to be the most stable gene. *16S* was the most stable gene for both sexes and *18S* was the most stable gene for RNAi experiments. The overall order based on the Δ*C*_*t*_ method, from the most-to-least stable reference genes is shown in Table 2.

### Comprehensive ranking of reference genes

**Figure 3:**
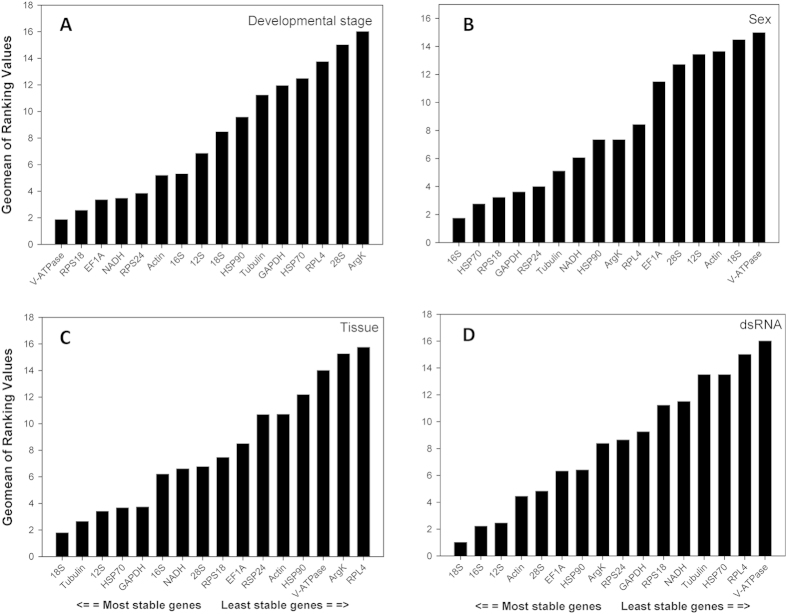
Stability of candidate reference genes expression under different treatments. A lower *Geomean* value indicates more stable expression according to *RefFinder*.

*RefFinder* is a comprehensive program that integrates all four above-mentioned software tools to rank the candidate reference genes based on their stability. The following rankings are listed in order of most-to-least stable reference genes. For the developmental stages, the comprehensive ranking was *V-ATPase*, *RPS18*, *EF1A*, *NADH*, *RPS24*, *Actin*, *16S*, *12S*, *18S*, *HSP90*, *Tubulin*, *GAPDH*, *HSP70*, *RPL4*, *28S*, *ArgK* (Fig. 3A). The overall ranking for sex was *16S*, *HSP70*, *RPS18*, *GAPDH*, *RPS24*, *Tubulin*, *NADH*, *HSP90*, *ArgK*, *RPL4*, *EF1A*, *28S*, *12S*, *Actin*, *18S*, *V-ATPase* (Fig. 3B). Different tissue types produced a ranking of *18S*, *Tubulin*, *12S*, *HSP70*, *GAPDH*, *16S*, *NADH*, *28S*, *RPS18*, *EF1A*, *RPS24*, *Actin*, *HSP90*, *V-ATPase*, *ArgK*, *RPL4* (Fig. 3C). For dietary RNAi treatments, the overall ranking was *18S*, *16S*, *12S*, *Actin*, *28S*, *EF1A*, *HSP90*, *ArgK*, *RPS24*, *GAPDH*, *RPS18*, *NADH*, *Tubulin*, *HSP70*, *RPL4*, *V-ATPase* (Fig. 3D).

### Quantitative analysis of candidate reference genes based on *geNorm*

**Figure 4:**
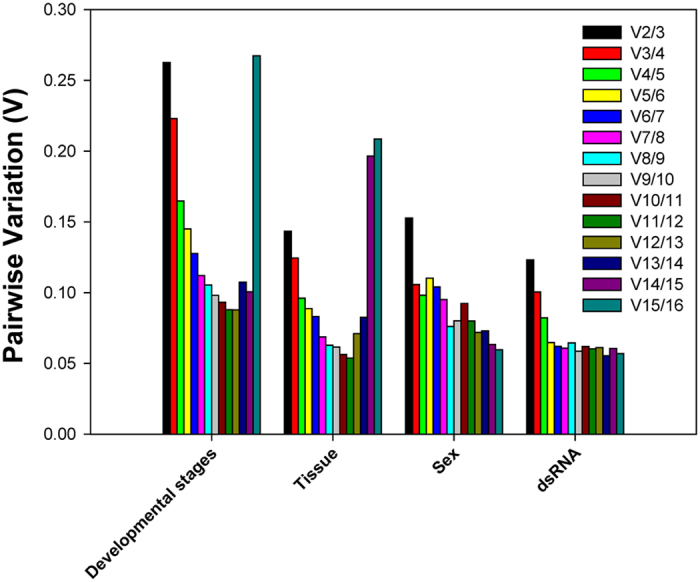
Pairwise variation (V) values in four experimental groups using *geNorm*.

Each experimental condition may demand a different set of requirements for normalizing the RT-qPCR data. The first V-value < 0.15 emerged at V5/6, suggesting that five reference genes are needed for reliable normalization throughout developmental stages (Fig. 4). In regard to tissue-specific and dietary RNAi experiments, the first V-value < 0.15 emerged at V2/3, suggesting that two reference genes are necessary for the reliable normalization (Fig. 4). Based on the same principle, three reference genes are required for the reliable normalization of ladybeetle samples with different sex as the first V-value < 0.15 appeared at V3/4 (Fig. 4).

### Relative gene expression of *V-ATPase*

**Figure 5:**
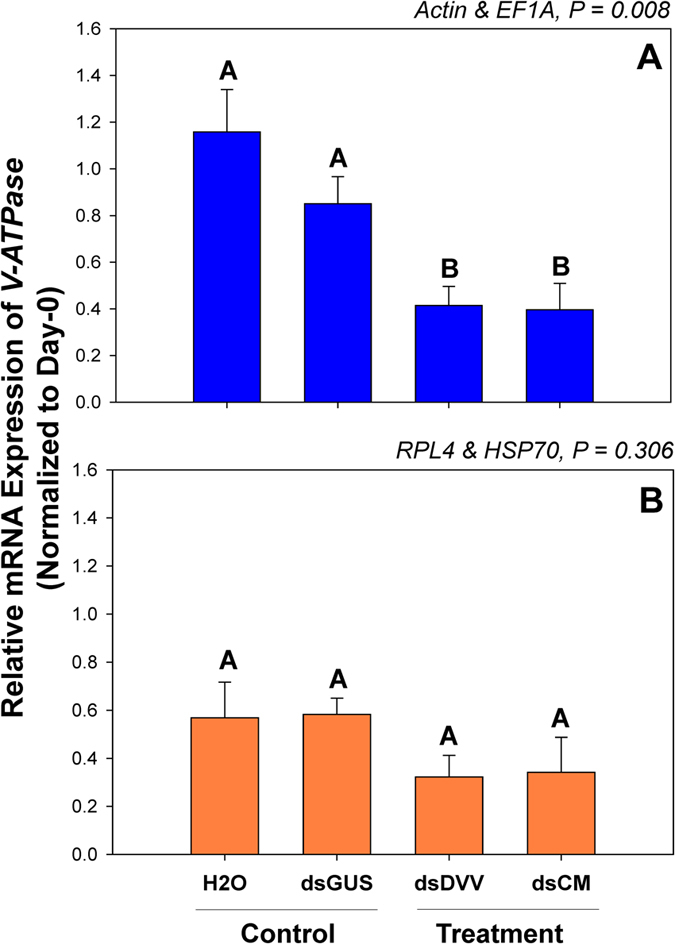
*Coleomegilla maculata V-ATPase* gene expression under dietary RNAi treatments. The relative mRNA expression levels of *V-ATPase* were normalized to the most suited (**A**, *Actin* and *EF1A*) and the least suited (**B**, *RPL4* and *HSP70*) reference genes, respectively. For dietary RNAi, ladybeetle larvae were exposed to an artificial diet containing 15% sugar solution and 4.0 μg/μl dsRNAs for two days (see Materials and Methods for details). The transcript levels of *V-ATPase* in newly emerged (0 day) untreated larvae were set to 1, and the relative mRNA expression levels in dsRNA-fed larvae were determined with respect to the controls. Values are means ± SE. Different letters indicate significant differences between the treatments and controls (*P* < 0.01).

The gene expression level of *V-ATPase* was significantly affected by the treatments when normalized to the two best stable non-rRNA reference genes *Actin* and *EF1A* (Fig. 5A) (F_3,8_ = 8.241, *P* = 0.008). Specifically, *V-ATPase* expression was significantly decreased at day 3 under the treatments of dsDVV and dsCM in comparison to the dsGUS and H_2_O controls (Fig. 5A). However, the gene expression level of *V-ATPase* was not affected by the treatments when normalized to the two least stable housekeeping genes *RPL4* and *HSP70* (Fig. 5B) (F_3,8_ = 1.423, *P* = 0.306). In this particular experimental setup, *V-ATPase* served as the target gene instead of the reference gene, which reflected by the highly varied expression levels under the dietary RNAi treatments (Fig. 3D).

## Discussion

Housekeeping genes, constitutively expressed to maintain basic cellular functions, are the conventional choice for a standardized reference^33^. Interestingly, there is, in fact, no "universal" reference gene that is stably expressed and applicable for all cell and tissue types across various experimental conditions^42^,^44^,^45^,^46^,^47^,^48^,^49^. Therefore, each candidate reference gene should be evaluated under specific experimental conditions^42^,^50^. Our results demonstrate that the suitable reference genes can be different in response to diverse biotic and abiotic conditions (Table 2; Fig. 3). For example, *GAPDH* was stably expressed in *C. maculata* under the tissue- and sex-specific conditions; however, its expression was highly variable among different developmental stages. This is consistent with the results from the convergens ladybeetle, *Hippodamia convergens* (Coleoptera: Coccinellidae), in which the expression of *GAPDH* was stable among different tissue types and sexes, but variable across different developmental stages^45^.

RT-qPCR is arguably the most widely used molecular technique for the detection and quantification of nucleic acids^50^. However, it is far from being a “gold standard” because of the lack of transparency, standardization and technical/quality controls^38^. Hellemans and Vandesompele^39^ estimated the average difference in expression level of a gene of interest after normalization with any of two randomly selected non-validated reference genes is between 3 and 6-fold among 10–25% of the case studies. Such inconsistency makes it impossible to draw a conclusion with biological or clinical relevance. To avoid biased normalization, more and more researchers have started to embrace the idea of using multiple reference genes to analyze gene expression^42^,^44^,^45^,^46^,^47^,^48^,^49^.

Determination of the optimal number of reference genes usually produces a trade-off between accuracy and practicality. In this study, five reference genes are required for reliable normalization under different developmental stages. In comparison, no more than three reference genes were required for reliable normalization under different sex, tissue types and dietary RNAi treatments. Metamorphosis has significant impact on the cellularity and consequently gene expression across the developmental stage. For examples, the *C*_*t*_ value of *ArgK* was approximately 27 from egg to the fourth instar larva, whereas *C*_*t*_ value increased to 35 at pupa and adult stage. Similarly, *GAPDH* had a *C*_*t*_ value of 27 at the pupa stage, whereas it decreased to 23 at the other stages.

Our analyses demonstrate a dynamic shift in gene expression levels when normalized to reference genes that were determined to be the most and least suitable for a given treatment conditions (Fig. 5A,B). This provides a case-specific framework for selecting the most appropriate genes for normalization, as comparative measurements can yield varying results when using different gene sets to normalize data. Our study is consistent with previous studies showing how the variability in reference gene expression under variable experimental conditions can statistically affects study outcomes, thus strongly supporting the argument for reference gene validation prior to their use experimentally^51^,^52^,^53^.

The mRNA expression level of *V-ATPase* in *C. maculata* was apparently affected by dietary RNAi treatments. *V-ATPase* expression was significantly reduced under the dsDVV and dsCM treatments compared to the dsGUS and H_2_O controls (Fig. 5A). *Coleomegilla maculate,* a conventional NTO surrogate species which serves as a biological control agent, seems to be susceptive to a systemic exposure to the ingested dsRNAs. As a sequence-specific gene silencing tool, RNAi has a great potential in agricultural applications, either through crop improvements or pest/disease controls. Before this novel pest control strategy can be regulated/commercialized, the ecological risk assessment of RNAi-based controls on NTOs must be preceded. Our study provides a road map for future investigations on the risk assessment of RNAi-based gene silencing biotechnologies, including RNAi insecticides and transgenic RNAi crops.

In summary, expression profiles of 16 candidate reference genes under four experimental conditions (different tissue types, developmental stages, sex, and dietary RNAi) were investigated using five readily available algorithms (*geNorm*, *NormFinder*, *BestKeeper*, Δ*C*_*t*_ method, and *RefFinder*). A suite of reference genes were specifically recommended for each experimental condition. These combined results reaffirm that there is no single universal reference gene suitable for all conditions, and reference genes can respond differently to various experimental conditions. This study represents the critical first step to establish a standardized RT-qPCR protocol for the functional genomics research in a ladybeetle *C. maculate*. Furthermore, it lays the foundation for conducting ecological risk assessment of RNAi-based gene silencing biotechnologies on non-target organisms; in this case, a key predatory biological control agent.

## Materials and Methods

### Insect cultures

*Coleomegilla maculata* (Coleoptera: Coccinellidae) was collected from cardoon, *Cynara cardunculus,* at the University of Kentucky in August, 2014. Larvae and adults were maintained in the laboratory and provisioned with pea aphids, *Acyrthosiphon pisum,* at 23 ± 0.5 °C, 16L: 8D photoperiod, and 50% relative humidity. Pea aphid clones were kindly provided by Dr. John Obrycki (University of Kentucky), and were maintained at 20–28 °C on fava bean seedlings, *Vicia faba* (Fabales, Fabaceae), in a greenhouse.

### Experimental conditions

#### Biotic factor

The different developmental stages included eggs, all four larval instars (collected at the first day of each instar), pupae, and adults (including both females and males). Tissue types, including head, gut, and carcass (the remaining tissues that removed head and viscera) were dissected from various instars of *C. maculate* larvae. For different sex, one adult female and male were collected, respectively.

#### Abiotic factor

For dietary RNAi treatments, the first-instar larvae were fed with an artificial diet containing 15% sucrose solution mixed with chemically synthesized dsRNAs from 1) a target species, the western corn rootworm, *D. v. virgifera* (dsDVV, Forward: TAATACGACTCACTATAGGGAGAGCTCTTTTCCCATGTGTAC; Reverse: TAATACGACTCACTATAGGGAGAGCATTTCAGCCAAACG), and 2) a NTO, *C. maculate* (dsCM, Forward: TAATACGACTCACTATAGGGAGATCTCTTTTCCCATGT; Reverse: TAATACGACTCACTATAGGGAGAGCATCTCGGCCAGAC). The molecular target here is *V-ATPase subunit A,* an energy related housekeeping gene. Controls included an exogenous control gene *β-glucuronidase* from bacteria (dsGUS, Forward: TAATACGACTCACTATAGGGAGAGGGCGAACAGTTCCTGATTA; Reverse: TAATACGACTCACTATAGGGAGAGGCACAGCACATCAAAGAGA), and H_2_O, the vehicle control. At the beginning of the experiment, *C. maculata* neonates that hatched in less than 24 hours were kept individually in each petri dish. Each neonate was provisioned with a 2 μl droplet containing 1 μl of dsRNA (8 μg/μl) and 1 μl of 30% sucrose solution on a daily basis. For the first two days, a total of 16 μg of dsRNA were provided to each neonate. On day-3, five individuals from each treatment were collected as one sample for the subsequent RT-qPCR analysis.

For the developmental stage, a total of 15 eggs were collected as one biological replicate, while one pupa was collected, individually, as one replicate. For the remaining developmental stages, and all other biotic and abiotic conditions, approximately five individuals were collected for each treatment, and each experiment was repeated three times independently. All collected samples were flash frozen in liquid nitrogen and stored at −80 °C in 1.5 ml centrifuge tubes. All the experiments were conducted at 23 °C with a photoperiod of 16: 8 (L: D).

### Total RNA extraction and cDNA synthesis

Total RNA was extracted using TRIzol reagent (Invitrogen, Carlsbad, CA) according to the methods described previously^44^,^45^. Total RNA was dissolved in 20–100 μl ddH_2_O and the concentration was quantified using a NanoDrop 2000c Spectrophotometer. Results for samples are as follows: eggs (367.7 ± 267.7 ng/μl), the first instar larvae (383.3 ± 164.8 ng/μl), the second instar larvae (424.3 ± 111.78 ng/μl), the third instar larvae (1037.0 ± 410.1 ng/μl), the fourth instar larvae (970.1 ± 8.46 ng/μl), pupae (1005.3 ± 51.4 ng/μl), adults (977.3 ± 345.1 ng/μl), heads (225.8 ± 8.6 ng/μl), carcasses (239.9 ± 60.1 ng/μl), and guts (233.7 ± 34.9 ng/μl). The OD260/280 ratio of all samples was between 1.9 and 2.1. First-strand cDNA was synthesized from 0.5 μg of total RNA using the M-MLV reverse transcription kit (Invitrogen, Carlsbad, CA) with a random N primer according to the manufacturer’s recommendations. The cDNA was diluted 10-fold for the subsequent RT-qPCR analyses.

### Candidate reference genes and primer design

A total of 16 candidate reference genes commonly used in RT-qPCR analyses in other insect species were selected (Table 1). Primers for *12S*, *16S*, *18S*, and *28S* were designed based on the sequences obtained from NCBI. For the other seven genes including *Tubulin*, *RPS24*, *HSP70*, *HSP90*, *NADH*, *RPS18*, and *RPL4* genes, primers were designed based on the sequences from a transcriptome of *C. maculate*^43^ (Table S2). For the *ArgK*, *EF1A*, *GAPDH*, *Actin*, and *V-ATPase* genes, degenerate primers were designed using CODEHOP (http://blocks.fhcrc.org/codehop.html) according to conserved amino acid residues among Coleoptera species (Table S1). Conditions for PCR amplifications have been described previously^44^,^45^. PCR products were cloned into the pCR4-TOPO vector (Invitrogen, Carlsbad, CA), and sequenced. After the identities of these reference genes were confirmed (Table S2), primers for the subsequent RT-qPCR analyses were designed online, https://www.idtdna.com/Primerquest/Home/Index.

### Reverse transcriptase-quantitative polymerase chain reaction (RT-qPCR)

The information regarding RT-qPCR analysis has been described previously^44^,^45^. In brief, gene-specific primers (Table 1) were used in PCR reactions (20 μl) containing 7.0 μl of ddH_2_O, 10.0 μl of 2×SYBR Green MasterMix (BioRad), 1.0 μl of each specific primer (10 μM), and 1.0 μl of first-strand cDNA template. The reactions were set up in 96-well format Microseal PCR plates (Biorad) in triplicates. Reactions were performed in a MyiQ single Color Real-Time PCR Detection System (BioRad). The standard curve and PCR efficiency of each candidate gene were constructed and calculated according to previously described methods^44^,^45^.

### Data analysis

One way ANOVA was used to compare the gene expression of *V-ATPase* under each dietary RNAi treatments. Stability of the 16 candidate reference genes were evaluated by algorithms *geNorm*^33^, *NormFinder*^54^, *BestKeeper*^55^, and the Δ*C*_*t*_ method^56^. Finally, *RefFinder* (http://www.leonxie.com/referencegene.php), a comprehensive software platform integrating all four algorithms, provided an overall ranking of the stability/suitability of these candidates^57^. Pairwise variation (V), as determined by *geNorm,* is an index for determining the optimal number of reference genes for accurate RT-qPCR normalization. A cut-off value for pairwise variation of 0.15 was recommended by Vandesompele *et al.* (2002)^33^. Beginning with two genes, this algorithm continuously adds another gene and recalculates the normalization factor ratio. If the added gene does not increase the normalization factor ratio over the proposed 0.15 cut-off value, the starting pair of genes is considered sufficient for normalizing data, otherwise, more genes should be incorporated.

## Additional Information

**How to cite this article**: Yang, C. *et al.* Selection of reference genes for RT-qPCR analysis in a predatory biological control agent, *Coleomegilla maculata* (Coleoptera: Coccinellidae). *Sci. Rep.* **5**, 18201; doi: 10.1038/srep18201 (2015).

## Supplementary Material

Supplementary Information

## Figures and Tables

**Table 1:** Primers used for RT-qPCR.

Gene	Primer sequences (5′–3′)	Length (bp)	Efficiency (%)	R^2^	Linear regression equation
*12S*	F:CGATAATCCACGATGGAATTTACTTTAG	140	98.0	0.9993	y=−3.3709x + 13.904
	R:CCCTTTCTTCTTTAGTATAAACTTCACC				
*28S*	F:ACCCGAAAGATGGTGAACTATG	101	96.3	0.9996	y=−3.4152x + 10.193
	R: CCAGTTCCGACGATCGATTT				
*18S*	F:AAGACGGACAGAAGCGAAAG	100	96.6	0.9993	y=−3.407x + 11.76
	R: GGTTAGAACTAGGGCGGTATCT				
*16S*	F:TTGAAGGGCCGCAGTATTT	99	98.5	0.9998	y=−3.3578x + 16.683
	R: AAGAAAGTCGTTCCCTCATCAA				
*EF1A*	F: TGAATTCGAAGCCGGTATCTC	92	105.3	0.9976	y=−3.2011x + 19.908
	R:CGCCGACAATGAGTTGTTTC				
*ArgK*	F:TCCGTTCAACCCATGTCTAAC	96	99.6	0.9993	y=−3.3312x + 22.235
	R: GTTCCTTTCAGTTCTCCATCCA				
*Actin*	F: CTTCCCGACGGTCAAGTTATC	93	101.1	0.9998	y=−3.2973x + 19.264
	R: GCAGGATTCCATACCCAAGAA				
*V-ATPase*	F: TTGACTGGAGGCGACATTTAC	113	104.4	0.9990	y=−3.2205x + 24.586
	R: CTTCCAGGTTCGGCTATGTATG				
*Tubulin*	F: GGTATCAATTACCAGCCACCA	144	99.2	0.996	y=−3.3426x + 22.26
	R: CTTGGCGTACATGAGATCGAA				
*GAPDH*	F: AACTGCTTGGCTCCGTTAG	107	98.6	0.9992	y=−3.3571x + 21.55
	R: CCATCGACAGTCTTCTGAGTTG				
*RPS24*	F: CCAGGACAACCATCGGTTAAA	93	101.1	0.9993	y=−3.2979x + 23.553
	R: GAAGCCGAATACGAAGCATACA				
*HSP70*	F: GCCGATGCGGAGAAGTATAAAG	100	99.4	0.9976	y=−3.3361x + 22.878
	R: CGGCTTGCTTGAGTTGGAATA				
*HSP90*	F: GTTGAATCGCCCTGTTGTATTG	105	96.5	0.9982	y=−3.409x + 24.273
	R: GTAACCCATTGTGGACGTATCT				
*NADH*	F: TCTGTTAGCTTTCATCCCATTGA	96	99.5	0.9983	y=−3.3345x + 18.3
	R: ATTGAGGCTGTAGCTTGTACTAAA				
*RPS18*	F: TACACCTTTGATCGCTGTGAG	108	99.9	0.9947	y=−3.3259x + 23.545
	R: GGCTCTGGTCATTCCAGATAAG				
*RPL4*	F: TGGAACCCTTGGAGTTTGTT	101	99.4	0.9942	y=−3.3306x + 27.864
	R: TGTACGACCACGCTGTATTG				

**Table 2:** Stability of reference gene expression under four experimental conditions.

Experimental conditions	Reference gene	*geNorm*		*Normfider*		*BestKeeper*		Δ*Ct*	
		Stability	Rank	Stability	Rank	Stability	Rank	Stability	Rank
Developmental stage	*V-ATPase*	0.909	3	0.399	1	0.548	3	1.277	1
	*12S*	1.060	8	0.879	9	0.837	9	1.400	3
	*16S*	1.010	6	0.756	7	0.822	8	1.362	2
	*18S*	1.035	7	0.760	8	0.892	10	1.437	8
	*Actin*	0.980	5	0.687	4	0.646	5	1.431	6
	*EF1A*	0.943	4	0.703	5	0.476	1	1.409	5
	*28S*	1.315	14	1.621	15	1.514	15	1.953	15
	*GAPDH*	1.143	11	1.069	11	1.188	14	1.556	11
	*RSP24*	0.720	1	0.723	6	0.606	4	1.476	9
	*RPS18*	0.720	1	0.666	3	0.522	2	1.434	7
	*NADH*	0.813	2	0.651	2	0.665	6	1.402	4
	*HSP90*	1.089	9	0.993	10	0.737	7	1.567	12
	*HSP70*	1.177	12	1.165	13	0.907	11	1.621	13
	*RPL4*	1.252	13	1.457	14	1.045	13	1.895	14
	*Tubulin*	1.117	10	1.071	12	0.935	12	1.540	10
	*ArgK*	1.694	15	4.263	16	3.690	16	4.348	16
Tissue	*V-ATPase*	0.816	13	0.917	14	1.114	14	1.457	14
	*12S*	0.515	3	0.153	1	0.491	11	1.007	3
	*16S*	0.600	6	0.518	7	0.426	6	1.077	5
	*18S*	0.540	4	0.321	2	0.185	1	1.000	1
	*Actin*	0.635	8	0.659	11	0.515	12	1.153	11
	*EF1A*	0.692	11	0.483	6	0.456	8	1.126	9
	*28S*	0.618	7	0.721	13	0.255	2	1.147	10
	*GAPDH*	0.443	1	0.572	8	0.361	4	1.086	6
	*RSP24*	0.656	9	0.705	12	0.458	9	1.169	12
	*RPS18*	0.674	10	0.465	5	0.438	7	1.110	8
	*NADH*	0.568	5	0.580	9	0.382	5	1.108	7
	*HSP90*	0.743	12	0.622	10	0.798	13	1.254	13
	*HSP70*	0.469	2	0.332	3	0.461	10	1.007	2
	*RPL4*	1.396	15	3.331	16	2.681	15	3.419	16
	*Tubulin*	0.443	1	0.453	4	0.377	3	1.049	4
	*ArgK*	1.107	14	3.049	15	2.687	16	3.154	15
Sex	*V-ATPase*	0.990	15	0.936	14	0.683	14	1.162	16
	*12S*	0.966	14	0.887	12	0.736	15	1.123	12
	*16S*	0.718	8	0.425	1	0.263	1	0.849	1
	*18S*	0.938	13	0.947	15	0.809	16	1.129	13
	*Actin*	0.867	11	0.963	16	0.647	12	1.151	15
	*EF1A*	0.903	12	0.880	11	0.612	11	1.113	11
	*28S*	0.824	10	0.921	13	0.656	13	1.130	14
	*GAPDH*	0.636	6	0.530	3	0.391	2	0.888	4
	*RSP24*	0.261	1	0.607	7	0.484	6	0.907	6
	*RPS18*	0.564	5	0.526	2	0.416	3	0.872	3
	*NADH*	0.688	7	0.603	6	0.437	4	0.912	7
	*HSP90*	0.485	4	0.651	8	0.534	8	0.926	9
	*HSP70*	0.261	1	0.579	4	0.503	7	0.871	2
	*RPL4*	0.761	9	0.724	10	0.452	5	1.006	10
	*Tubulin*	0.403	2	0.580	5	0.562	9	0.890	5
	*ArgK*	0.440	3	0.652	9	0.565	10	0.913	8
dsRNA	*V-ATPase*	0.786	15	0.900	16	0.784	16	1.050	16
	*12S*	0.293	1	0.294	3	0.279	4	0.634	3
	*16S*	0.362	2	0.257	2	0.203	2	0.628	2
	*18S*	0.293	1	0.147	1	0.187	1	0.586	1
	*Actin*	0.407	3	0.340	4	0.305	6	0.649	4
	*EF1A*	0.492	7	0.343	5	0.362	8	0.666	5
	*28S*	0.436	4	0.428	6	0.221	3	0.696	6
	*GAPDH*	0.559	9	0.569	9	0.449	9	0.794	9
	*RSP24*	0.527	8	0.477	7	0.480	11	0.729	8
	*RPS18*	0.595	10	0.681	12	0.461	10	0.868	12
	*NADH*	0.633	11	0.668	11	0.547	12	0.857	11
	*HSP90*	0.450	5	0.504	8	0.302	5	0.726	7
	*HSP70*	0.705	13	0.748	13	0.688	14	0.925	13
	*RPL4*	0.748	14	0.870	15	0.713	15	1.022	15
	*Tubulin*	0.673	12	0.786	14	0.680	13	0.944	14
	*ArgK*	0.469	6	0.616	10	0.344	7	0.801	10
