# Supplementary material for: Selection of reference genes for RT-qPCR analysis in a predatory biological control agent, Coleomegilla maculata (Coleoptera: Coccinellidae)
Source: Sci Rep. 2015 Dec 10;5:18201. doi: 10.1038/srep18201 (PMC4674751; doi:10.1038/srep18201)
Supplement: Supplementary Information [file srep18201-s1.doc]

**Supplementary information**

### Selection of reference genes for RT-qPCR analysis in a predatory biological control agent, *Coleomegilla maculata* [(Coleoptera: Coccinellidae)](http://journals.cambridge.org/abstract_S0008347X00018629)

Chunxiao Yang^1,2†^, Huipeng Pan^2†^, Jeffrey Edward Noland^2^, Deyong Zhang^1^, Zhanhong Zhang^3^, Yong Liu^1*^, Xuguo Zhou^2*^

**Figure S1.The agrose gel electrophoresis of the 16 candidate reference genes.** M, EZ Load^TM^ 100 bp Molecular Ruler; Templates in the PCR reactions were as follows: 1) *12S*; 2) *28S*; 3) *18S*; 4) *16S*; 5) *EF1A*; 6) *ArgK*; 7) *Actin*; 8) *V-ATPase*; 9) *Tubulin*; 10) *GAPDH*; 11) *RPS24*; 12) *HSP70*; 13) *HSP90*; 14) *NADH*; 15) *RPS18*; 16) *RPL4*.

**Figure S2. Standard curves of the 16 candidate reference genes.**

**Table S1. Degenerate primers used for RT-qPCR**

**The sequence information of these 12 reference genes including *Tubulin*, *RPS24*, *HSP70*, *HSP90*, *NADH*, *RPS18*, *RPL4*, *Actin*, *EF1A*, *GAPDH*, *ArgK*, *V-ATPase***

**Figure S1.The agrose gel electrophoresis of the 16 candidate reference genes.**

**Figure S2. Standard curves of the 16 candidate reference genes.**

**Table S1. Degenerate primers used for RT-**qPCR

| Gene | Primer sequences (5’-3’) |
| --- | --- |
| *V-ATPase* | F: ACGAGCTGACCCAGTCCATHTAYATH |
|  | R: CACGTTGTAGCCCATATCGCKRAART |
| *GAPDH* | F: AACGCCTCCTGCACCACNAAYTGYYT |
|  | R: CGGGCACGCGGAANGCCATNCC |
| *Actin* | F: CGACATGGAAAAGATCTGGCAYCAYAC |
|  | R: TCGGTCAGCGATACCAGGRTACATNGT |
| *ArgK* | F:CCTGTTCGACCCTATCATCGARGAYTAYCA |
|  | R:GTCGTAGATACCACCTTCAGCTTCNGTRTGYTC |
| *EF1A* | F: TCGACATCGCTCTGTGGAARTTYGARAC |
|  | R: GTACCGATACCACCGATTTTGTANACRTCYTG |

**The sequence information of these 12 reference genes including *Tubulin*, *RPS24*, *HSP70*, *HSP90*, *NADH*, *RPS18*, *RPL4*, *Actin*, *EF1A*, *GAPDH*, *ArgK*, *ATPase***

>AbA_Locus_32_Transcript_6/9_Confidence_2.000_Length_1302 *a-tubulin* (*Tubulin*)

TCTTCCACCCCGAACAACTCATCACCGGCAAAGAGGACGCCGCTAACAACTACGCCCGTG

GTCATTACACCATCGGAAAAGAGATTGTAGACTTGGTTTTGGATCGTATCCGTAAGTTGG

CGGATCAATGTACAGGATTGCAAGGATTCTTGATCTTCCACCCCGAACAACTCATCACCG

GCAAAGAGGACGCCGCTAACAACTACGCCCGTGGTCATTACACCATCGGAAAAGAGATTG

TAGACTTGGTTTTGGATCGTATCCGTAAGTTGGCGGATCAATGTACAGGATTGCAAGGAT

TCTTGATCTTCCACTCCTTCGGCGGTGGTACCGGATCCGGATTCACTTCCCTTTTGATGG

AAAGGTTATCCGTGGATTATGGAAAGAAATCGAAATTGGAATTCGCCATATATCCGGCAC

CTCAGGTATCTACCGCTGTGGTAGAACCATACAACTCGATCCTGACCACCCACACCACCC

TGGAACACTCTGACTGCGCCTTCATGGTAGACAACGAGGCCATCTACGATATCTGCAGAC

GTAACTTGGACATCGAACGTCCTACATACACCAACTTGAACAGGCTGATCGGTCAAATCG

TGTCATCGATTACGGCTTCCCTGAGGTTCGACGGCGCCCTAAACGTCGATCTTACCGAAT

TCCAGACCAACTTGGTGCCGTACCCGCGTATCCATTTCCCACTGGTCACCTACGCGCCAG

TAATTTCCGCCGAGAAGGCCTACCACGAGCAGTTGTCCGTCGCAGAAATCACCAATGCCT

GCTTCGAACCAGCCAACCAGATGGTAAAATGCGATCCGCGTCACGGCAAATACATGGCTT

GCTGTATGTTGTACAGAGGTGACGTCGTACCAAAGGACGTTAATGCAGCCATCGCCACCA

TCAAGACCAAACGTACCATCCAGTTCGTTGACTGGTGTCCCACCGGATTCAAGGTCGGTA

TCAACTACCAGCCACCAACCGTCGTGCCAGGCGGCGACCTCGCTAAGGTACAGAGGGCCG

TGTGCATGTTGTCCAACACCACCGCCATCGCCGAGGCTTGGGCCAGGCTCGATCACAAGT

TCGATCTCATGTACGCCAAGAGAGCCTTCGTTCATTGGTACGTGGGTGAGGGTATGGAAG

AAGGTGAGTTCTCTGAGGCCCGTGAAGATTTGGCCGCTCTGGAGAAGGACTACGAAGAGG

TTGGCATGGATTCTGGAGAGGGTGAGGGTATGGAAGAAGGTGAGTTCTCTGAGGCCCGTG

AAGATTTGGCCGCTCTGGAGAAGGACTACGAAGAGGTTGGCA

>AbA_Locus_8_Transcript_27/41_Confidence_0.000_Length_771 *40S ribosomal protein S24* (*RPS24*)

TTGTTTCCTAGTTTGTTGTTTCTTTTTGGTACCCCTAACTTTCTTCATTCTGTTCTTCCT

TTCTTTACGTTGTTTCCTAGTTTGTTGTTTCTTTTTGGTACCCCTAACTTTCTTCATTCT

GTTCTTCCTTTCTTTACGTTGTTTCCTAGTTTGTTGTTTCTTTTCATACAAACCGTGCCT

GGCTAACCTGTGTTTCGGTTCGAACTTTTTGGCGAAATCTAAGGTGTCGTAGATTAAGGC

GAATCCAGTGGATTTACCGCCTCCAAAGTTAGTTCTGAAGCCGAATACGAAGCATACATC

GGGAGTTACTTTGTACATTTTGGCCAACTTTTCCCTAATATCGGTTTTTTTAACCGATGG

TTGTCCTGGATGGAGAACATCAACGACCATTTGTTTGCGACACAACAATCTGTTGGTCAT

GAATTTCCTAGTGCGAATGGTAGCAGTTCCTTCCGCCATTTTGAGAACACCAAGAGCACT

AGGAAATTCATGACCAACAGATTGTTGTGTCGCAAACAAATGGTCGTTGATGTTCTCCAT

CCAGGACAACCATCGGTTAAAAAAACCGATATTAGGGAAAAGTTGGCCAAAATGTACAAA

GTAACTCCCGATGTATGCTTCGTATTCGGCTTCAGAACTAACTTTGGAGGCGGTAAATCC

ACTGGATTCGCCTTAATCTACGACACCTTAGATTTCGCCAAAAAGTTCGAACCGAAACAC

AGGTTAGCCAGGCACGGTTTGTATGAAAAGAAACAACAAACTAGGAAACAA

>AbA_Locus_27_Transcript_9/32_Confidence_0.000_Length_894 *heat shock protein 70* (*HSP70*)

TCACAACGTACGCAGACAACCAACCTGCGGTAACCATCCAAGTGTTCGAAGGCGAAAGAG

TTATGACCAAGGATAACAACCTACTTGGCACCTTCGACTTGACCGGAATACCCCCAGCTC

CTCGTGGAGTCCCCAAAATTGAAGTAACATTCGATTTGGACGCCAACGGAATATTGAATG

TATCAGCAAAGGACACTAGCTCTGGCAATTCCAAAAACATCACCATCAAAAACGATAAAG

GAAGGTTATCAGCTCCGGCAATTCCAAAAACATCACCATCAAAAACGATAAAGGAAGGTT

ATCCCAGAGAGACATCGATCAAATGGTGGCCGATGCGGAGAAGTATAAAGAAGAAGATGA

GAAGCAAAAACAAAGAATCGAAGCTCGCAACAAACTGGAGGCTTACGTATTCCAACTCAA

GCAAGCCGTACAGGATTGCGGAAGCAAACTGAGCAACGAAGACAAAGCTACTGTGGAAAG

AGAATGTCAGAATTGCCTGCAGTGGCTCGACTCCAATACTTTGGCTGAAAAGGATGAATA

CGAAGATAAACAAAAACAACTTACATCAATTTGCAGTCCTATAATGGCCAAATTATATGG

AGGTGCTCAGAATAATACCAACTTTGGCGGTATGCCAGGTGGCTGTGGACAGCAGGCCGG

CGAAGGTTTTGGCGGCAGACAAGGAGGACCTACCATTGAAGAAGTCGATTAAATTAATGC

AGCATCACTGATTCCCTCTTCATCATCTCGGCATTTCCTGCATTGGTTTACTCGATGGAG

AGTGGATTATATTGAAGAAGTCGATTAAATTAATGCAGCATCACTGATTCCCTCTTCATC

ATCTCGGCATTTCCTGCATTGGTTTACTCGATGGAGAGTGGATTATATTGAAGA

>TC_Locus_17_Transcript_1/3_Confidence_1.000_Length_4618 *heat shock protein 90 (HSP90)*

TTTAAATTGTATGTTATTTTATTCTCAATATTCAACAGTTATCAGTACAAGGGCAACTCT

GTTGGAACACAGCCAAAACTTATTTTTTCGAGATAGAAAATATAAAAAATAACTTATAGG

AATGAAGAGAACATACTAAAACACATGTTCAATTAATACATAAAAGAGAGTAGTATTCGA

GAAATTAATCAACTTCTTCCATTCTTGAAGCATCTTCAGAATCGCCTGCTTCAGCAGCTG

GAGCAGCTTCTGTAGATGGTTCTTCAACTACCATTGATTCTTCTTCATCAATACCCAAAC

CCAACTTGATCATTCTGTAGATCCTGGAAGCATGTACTTGTGGTTCATCCAAAGTGAATC

CTGAGCTGAGAAGTGAAGTTTCGAATAACAAAATTACAAGATCCTTGACAGCCTTATCAT

TCTTGTCAGCTTCAGCCTTCTGTCTCAAGTTTTCAATGATTGGATGGTCTGGATTGATTT

CAAGATGTTTCTTCGCAGACATGTAACCCATTGTGGACGTATCTCTGAGAGCTTGTGCTT

TCATGATACGCTCCATGTTAGCGGTCCATCCATATTGAGAAGTTACAATACAACAGGGCG

ATTCAACCAACCTATTTGATACGACCACCTTCTCTACTTTATTGTCCAAAATGCTCTTGA

TAACTTTGCATAATCCTTCGAATTTAGCTTTATCTTCTTCGCGTTTCTTCTTTTCTTCTT

CATCTTCAGGCAACTCCAAACCTTCTTTAGTCACAGAAACCAATGTTTTGCCATCATATT

CTTTCAGTTGTTGTACGACATATTCATCGATGGGCTCAGTCATATAAACAACTTCAAATC

CTCGCTTCTTGACACGCTCAACAAATACTGAATTGGCAACCTGCTCTTTGCTCTCACCAG

TAAGGTAGTAAATGCTCTTTTGGTTTTGTTTCATTCTGCTAACATAATCTTTAAGAGAAC

ATGCTTCATCTCCACTGGCTGAAGTATGGTAACGGAGGAATTCAGCTAATTTAGATCGAT

TGGCGGAGTCCTCGTGAATGCCCAGTTTAAGATTCTTGGAGAATTGTTCATAGAATTTCT

TGAAGTTATCTTTGTCTTCTGTCAACTCCTCAAACAATTCCAAGCATTTCTTAACCAAGT

TCTTGCGAATAACCTTAAGAATTTTGTTTTGTTGTAACATTTCTCTGGAAATGTTCAAAG

GTAAATCTTCAGAGTCCACAACACCCTTGATGAAATTCAAATACTCAGGAATGAGTTCAT

CGCAGTTATCCATAATGAATACTCTGCGTACGTACAATTTGATGTTATTCTTGCGCTTCT

TATTTTCAAAAAGATCAAATGGTACTCTGCGTGGAACAAATAATAGAGCTCTAAATTCCA

ATTGACCTTCCACGCTGAAGTGTTTGACAGCCAAATGGTCTTCCCAATCGTTGGTGAGAG

ATTTGTAGAATTCACCGTATTCTTCTTGAGAAATATCATCGGCATTTCTTGTCCAGATAG

GTTTAGTCTTGTTGAGCTCTTCATCTTCTGTGTATTTCTCTTTGATGGTCTTCTTTTTCT

TCTTCTTCTCTTTGTCTTCTTCCTCATCCTCTCCTACATCTTCAATCTTTGGTTTGTCAG

CATCTTCAGTTACCTCTTCATCTTTCTTCTCTTCTTCAGCCTCATCTTCACTCAATTCCT

TTTCACGTTCCTTTTCTACCAACAATTTGATAGGATAACCAATGAATTGGGAGTGCTTCT

TAACGATATCTTTGATTTTGTGTTCTTCCAAGAATTCTGCTTGATCTTCCTTGATATGGA

GAACGATCTTGGTACCTCTACCAAGTGGCTCCCCACTGTCGACACGTACTGTGAAACTAC

CACCAGCCGATGACTCCCAAATGTACTGTTCATCATCATTATTCTTTGAAACTACTGTTA

CTTTGTCGGCAACCAAGTAGGCGGAGTAGAAACCAACACCAAATTGACCAATCATGCTAA

TATCAGCTCCAGCTTGCAAAGCTTCCATGAAGGCCTTGGTTCCAGACTTGGCAATGGTAC

CCAAATTGTTGACTAAATCGGCCTTAGTCATACCAATACCAGTATCGATGATTGTTAGGG

TACCATCGTTCTTGTTTGGGATGATCTTGATGTAGAGTTCTTTTCCAGAATCCAAGCAGG

TAGGATTTGTCAGAGACTGATAACGGATTTTATCGAGAGCATCAGAAGAGTTTGAAATTA

ATTCTCTAAGGAAAATTTCCTTATTAGAATAGAAGGTGTTGATAATCAAACTCATCAACT

GAGCAATCTCAGCCTGGAAGGCGAAAGTTTCGACTTCCAGGCCAGGCTGAGATTGCTCAG

TTGATGAGTTTGATTATCAACACCTTCTATTCTAATAAGGAAATTTTCCTTAGAGAATTA

ATTTCAAACTCTTCTGATGCTCTCGATAAAATCCGTTATCAGTCTCTGACAAATCCTACC

TGCTTGGATTCTGGAAAAGAACTCTACATCAAGATCATCCCAAACAAGAACGATGGTACC

CTAACAATCATCGATACTGGTATTGGTATGACTAAGGCCGATTTAGTCAACAATTTGGGT

ACCATTGCCAAGTCTGGAACCAAGGCCTTCATGGAAGCTTTGCAAGCTGGAGCTGATATT

AGCATGATTGGTCAATTTGGTGTTGGTTTCTACTCCGCCTACTTGGTTGCCGACAAAGTA

ACAGTAGTTTCAAAGAATAATGATGATGAACAGTACATTTGGGAGTCATCGGCTGGTGGT

AGTTTCACAGTACGTGTCGACAGTGGGGAGCCACTTGGTAGAGGTACCAAGATCGTTCTC

CATATCAAGGAAGATCAAGCAGAATTCTTGGAAGAACACAAAATCAAAGATATCGTTAAG

AAGCACTCCCAATTCATTGGTTATCCTATCAAATTGTTGGTAGAAAAGGAACGTGAAAAG

GAATTGAGTGAAGATGAGGCTGAAGAAGAGAAGAAAGATGAAGAGGTAACTGAAGATGCT

GACAAACCAAAGATTGAAGATGTAGGAGAGGATGAGGAAGAAGACAAAGAGAAGAAGAAG

AAAAAGAAGACCATCAAAGAGAAATACACAGAAGATGAAGAGCTCAACAAGACTAAACCT

ATCTGGACAAGAAATGCCGATGATATTTCTCAAGAAGAATACGGTGAATTCTACAAATCT

CTCACCAACGATTGGGAAGACCATTTGGCTGTCAAACACTTCAGCGTGGAAGGTCAATTG

GAATTTAGAGCTCTATTATTTGTTCCACGCAGAGTACCATTTGATCTTTTTGAAAATAAG

AAGCGCAAGAATAACATCAAATTGTACGTACGCAGAGTATTCATTATGGATAACTGCGAT

GAACTCATTCCTGAGTATTTGAATTTCATCAAGGGTGTTGTGGACTCTGAAGATTTACCT

TTGAACATTTCCAGAGAAATGTTACAACAAAACAAAATTCTTAAGGTTATTCGCAAGAAC

TTGGTTAAGAAATGCTTGGAATTGTTTGAGGAGTTGACAGAAGACAAAGATAACTTCAAG

AAATTCTATGAACAATTCTCCAAGAATCTTAAACTGGGCATTCACGAGGACTCCGCCAAT

CGATCTAAATTAGCTGAATTCCTCCGTTACCATACTTCAGCCAGTGGAGATGAAGCATGT

TCTCTTAAAGATTATGTTAGCAGAATGAAACAAAACCAAAAGAGCATTTACTACCTTACT

GGTGAGAGCAAAGAGCAGGTTGCCAATTCAGTATTTGTTGAGCGTGTCAAGAAGCGAGGA

TTTGAAGTTGTTTATATGACTGAGCCCATCGATGAATATGTCGTACAACAACTGAAAGAA

TATGATGGCAAAACATTGGTTTCTGTGACTAAAGAAGGTTTGGAGTTGCCTGAAGATGAA

GAAGAAAAGAAGAAACGCGAAGAAGATAAAGCTAAATTCGAAGGATTATGCAAAGTTATC

AAGAGCATTTTGGACAATAAAGTAGAGAAGGTGGTCGTATCAAATAGGTTGGTTGAATCG

CCCTGTTGTATTGTAACTTCTCAATATGGATGGACCGCTAACATGGAGCGTATCATGAAA

GCACAAGCTCTCAGAGATACGTCCACAATGGGTTACATGTCTGCGAAGAAACATCTTGAA

ATCAATCCAGACCATCCAATCATTGAAAACTTGAGACAGAAGGCTGAAGCTGACAAGAAT

GATAAGGCTGTCAAGGATCTTGTAATTTTGTTATTCGAAACTTCACTTCTCAGCTCAGGA

TTCACTTTGGATGAACCACAAGTACATGCTTCCAGGATCTACAGAATGATCAAGTTGGGT

TTGGGTATTGATGAAGAAGAATCAATGGTAGTTGAAGAACCATCTACAGAAGCTGCTCCA

GCTGCTGAAGCAGGCGATTCTGAAGATGCTTCAAGAATGGAAGAAGTTGATTAATTTCTC

GAATACTACTCTCTTTTATGTATTAATTGAACATGTGTTTTAGTATGTTCTCTTCATTCC

TATAAGTTATTTTTTATATTTTCTATCTCGAAAAAATAAGTTTTGGCTGTGTTCCAACAG

AGTTGCCCTTGTACTGATAACTGTTGAATATTGAGAATAAAATAACATACAATTTAAA

>AbA_Locus_54_Transcript_2/5_Confidence_1.000_Length_952 *NADH dehydrogenase subunit 2* (*NADH*)

TTCTTTATTCGCTTTTTACTAAAACCTCTCTCGAACATCTGCCCTCAATTTCTAATTTTA

TTTTTAATTGTGCTATCCTTATAAAAATAGGAGCAGCTCCTATTTTTATAAGGATAGCAC

AATTAAAAATAAAATTAGAAATAAATCTGTTAGCTTTCATCCCATTGATTAACAGAGATA

ATTATAGTCAATCTGCTGAAGTATCTTTAAAATATTTTTTAGTACAAGCTACAGCCTCAA

TATTTATTATATTTGCATTTCTTTATTCGCTTTTTACTAAAACCTCTCTCGAACATCTGC

CCTCAATTTCTAATTTTATTTTTAATTGTGCTATCCTTATAAAAATAGGAGCAGCTCCTT

TTCATTTTTGATACCCCGAAGTATCTGAAGGTTTAAGATGAATTAATAATTTAATTTTAA

TGACATGACAAAAAATTGCCCCTATAATTTTACTTATATATAATTTTAAAATTAATAGAT

TTTTTTGCTTTATTATTATCATTTCTATAACAGTTAGAGGGCTAAAAAGATGAAATCAAA

CTAGGTTAAAAAAAATTTTAGCCCTATCTTCAATTAATCACATTGGATGAATAATAACGT

TGATATTCTTTAACCAATCTCTTTGATTATTTTATTTTTTATTCTATCTATTCATTTCTA

CTAATATAATTATAATCTTTGATAAATTTAAAATTAATAATATATACCAACTTCTAAATT

TATTTAATTTTAATAAATCAATAAAATTTTTTTTCTTTCTGAATTTTTTTTCTTTAGGAG

GTATCCCTCCTTTTCTTGGATTTTTCCCTAAATGACTAGCTATTAAAATCTTAATTAATA

ACAATATAAGATTACTAGCAATTTTAATGATTTTCTTAACATTATTAAGATTATTTATCT

ATATTCGAATTATAATAAAACCTTTAATGTTTAAAATTTCTGAAAAAAAAAA

>AbA_Locus_56_Transcript_1/2_Confidence_1.000_Length_774 *ribosomal protein S18* (*RPS18*)

GTAGTTTTGGTGTGTTGACCTCTTACATTGTCAAATTAAAATTACTTCTTCTTGGAGACA

CCCACAGTTCTACCACGTCTACCAGTAGTTTTGGTGTGTTGACCTCTTACATTGTCAAAT

TAAAATTACTTCTTCTTGGAGACACCCACAGTTCTACCACGTCTACCAGTAGTTTTGGTG

TGTTGACCTCTTACTCTCAGACCCCAGTAATGTCTCAGACCCCTGTGGGCTCGAATCTTC

TTCATCCTCTCCAAATCTTCACGAAGTTTAGAATCAAGTGCTGACGATGTCAACTGGGAG

TATTTACCATCGACAATGTCTTTTTGCCTGTTGAGGAACCAATCAGGGATTTTATATTGT

CTAGGATTGGACATAATGGTAATAATTTTTTCAACTTCCTCTTCGCTGCATTCTCCTGCT

CGTTTACGAAGATCAACATCAGCCTTCTTGCAAATAATGTTGGAGTATCTACGACCTACA

CCTTTGATCGCTGTGAGGGCGAACATGACATTCCTTTTTCCATCGATATTCGTACCGAGG

ATACGCAAAATGTGCTGAAAAACTTATCTGGAATGACCAGAGCCATTGTGACGAACGAAA

CTTCTCAACTTCCTCTTCGCTGCATTCTCCTGCTCGTTTACGAAGATCAACATCAGCCTT

CTTGCAAATAATGTTGGAGTATCTACGACCTACACCTTTGATCGCTGTGAGGGCGAACAT

GACATTCCTTTTTCCATCGATATTCGTACCGAGGATACGCAAAATGTGCTGAAA

>TB2_Locus_147_Transcript_79/90_Confidence_1.000_Length_1862 *ribosomal protein L4* (*RPL4*)

ACCTTGGTTGTATCCGTAGTGTTGTTGGCCATACCAGTAGTTTTCACCGTAAATGTTCTT

AGTGTATTGTGGAGCATATTTGTAGCCTTCGAAGAAGTGTTGGAATGGAACCCTTGGAGT

TTGTTGAAAGAAGTTGTTTTTGAAGAGGTAGTTCAATCCCAAGTATTCGTTATCGACGAA

TTGTTTCAATACAGCGTGGTCGTACACTCCATATTGTACCAAAAAGTTGTATAAAGCTTC

TTTGTTTACCAATTGTCTCAGAAGTACTTGGTTGTACACACCCTCAGTTTGGTACTGATA

CAAGATTTGGTCGAGGACGTTCTTGTAGAAGATTCCATATTGGACCATAACGTTCCTCCA

GTAAGGGTTGTAGATGATTTTTTATAAGGATTGTAGATGATTTTTTCTAAAGTTTCGTAG

CTGTAGACACCCTTGTTGAAGTAAGCGTTTCCGTAGTATTGGTTGTAGTAGTTTGTCCCA

AGATGTCGTATACGGAAATGTCATGGTTGATGAAAAAGTATGTACGTAGTTTGTCCCAAG

ATGTCGTATACGGAAATGTCATGGTTGATGAAAAAGTATTTCAACACTTGAGTATTGACC

AAACTGTGGAATAAGTAATGGTTGTAAACTCCTTCAGTCTTGTAAGTGTAGAGGATTTGA

TCGAGGACAACTTTGTCGAAGTATCCATGTTGGACGAACCAGGATCTGAGAGTTGGGTTG

TTGATGATTTCTACCAAGTATTCATAATCGAAGATACCTTCATGGTTGAAAACGAAGTTC

CACAATTTTTCATGAGAGTATTTTCCGTACATTTCCTGGTTGAACATTTGTCCTTGGATC

AAGACGTTCTTCAATACTTTTCTGTCAAAGATTTTCATCAAGATGTTGTTGTAGTAGTGA

ACATCGTATTGGTTGTAGTCTTGTCCATAAATTCCCCTTTGTTTGAAAAGTTTTCCGTAA

AAATCGACCCCTTTGTTTGAAAAGTTTTCCGTAAAAATCGAGTCCGTAAGTTTTCTGCTG

GGTCATTTTGTTCAATACTTCACCAATTTGTCCTTCGAAAGATTTTCCAGAGACCAGGTT

ATCCCATTTGGAGTCCTCAACGAGAGCTTTGAGCAGATACAAGACTTTGCCGTCGAAGTG

ACCGTCAAAAACAGCCTTTTGGACCTTGGTGAGAAGTTGTTGGTAAGCGTACTCATCAAC

GTGACCTAAATACTCCTTGAAGATGACATCGAAAACGTCGTGTTGACTGCCGTACACCTT

GTTGAAGGTGTTGTAGTCGAAGTGATACGTTTTGGTGAAGTCATTGGGTCCAGTTACTTC

GATTTCGATTTTTTTTTCCTGAGGTCCAAAATGCTTGGTGATGTAAATCTGCTTACCATG

TATACAGCGCGTCCGCTGATAAGTGTCTTCGACGAGAAGGCCAAGCCAGTTCCAGAAGCA

AATGTTGTTTTGCCTGCAGTATTTCGTGCTCCGATCAGGCCAGATGTTGTCTCTTTTGTA

CAACAGCAGGTTTCTATGAACCATAGGCAACCCTATTGTGTCAGTGAAAAAGCTGGTCAT

CAAACGTCTGCTGAGTCATGGGGTACTGGAAGAGCCGTTGCTCGTATTCCTCGTGTAAGA

GGTGGTGGTACCCACCGATCTGGTCAGGGTGCTTTCGGCAACATGTGTAGGGGAGGTCGC

ATGTTTGCACCTACTAAACCATGGCGTAGGTGGCACCGTAGGGTTAACATCAACCAGAGG

CGTTATGCTCTTGCTTCGGCTATAGCCGCTAGTGGCATACCAGCTTTGGTAATGAGCAAG

GGACATGCCATCGATCAAGTTCCTGAACTTCCTTTGGTCCGTTTCGACAAAGTACAAGAA

CT

> *arginine kinase* (*ArgK*)

CCTGTTCGACCCTATCATCGAAGATTATCATGGTGGATTCAAAAAGACCGATAAACATCCCCCAAGAGATTTTGGAGACGTCAGCATTTTTGGAAATTTGGATCCTGCTGGAGAGTACATCGTGTCCACACGTGTTCGATGTGGACGTTCTTTGGAGGGATATCCGTTCAACCCATGTCTAACAGAAGAACAGTACAAGGAAATGGAACAGAAAGTTTCATCAACATTGTCAGGACTGGATGGAGAACTGAAAGGAACATTCTATCCATTAACTGGAATGGACAAGGAAACTCAGCAGAAGTTGATTGATGATCACTTTTTGTTCAAAGAAGGCGACAGATTTTTGCAAGCCGCAAACGCTTGCAGATTTTGGCCAAGTGGTCGTGGAATATTCCACAACGATGCTAAAACCTTCTTAGTCTGGTGCAACGAAGAAGATCACTTGAGAATTATATCCATGCAAATGGGTGGCGATCTTGGAGAAGTTTACCGCCGATTGGTAACTGCAGTTAATGAAATCGAAAGACGTTTGCCATTTTCTCACAACGACAGGCTTGGTTTCCTCACTTTCTGTCCAACCAACTTGGGCACCACTGTCAGAGCCTCGGTGCACATAAAGGTACCAAAATTGGCCGCCAATAAGGCTAAGCTTGATGAGGTTGCCGGCAAGTTCAACCTTCAGGTTCGTGGGACACGTGGGGAGCATACCGAAGCTGAAGGTGGTATCTACGAC

> *elongation factor 1 α* (*EF1A*)

TCGACATCGCACTGTGGAAGTTCGAAACTTCAAAATACTACGTTACAATTATTGACGCCCCTGGACACAGAGATTTTATCAAGAACATGATCACAGGCACATCTCAGGCAGATTGTGCCGTATTGATTGTAGCAGCCGGTACCGGTGAATTCGAAGCCGGTATCTCGAAGAATGGACAAACTCGCGAGCACGCCCTTCTCGCCTTCACTTTGGGAGTGAAACAACTCATTGTCGGCGTTAACAAGATGGACTCCACCGAGCCACCTTACAGCGAGTCTCGTTTTGAGGAAATCAAGAAGGAAGTATCTTCGTACATCAAGAAGATCGGTTACAACCCGGCTGCTGTTGCTTTCGTGCCCATTTCCGGATGGCACGGAGACAACATGTTGGAGCCATCCACCAAGATGCCATGGTTCAAGGGATGGGCCATTGAACGTAAAGAAGGAAAAGCCGATGGTAAATGCTTGATCGAAGCACTCGATGCCATCCTTCCCCCATCTCGTCCAACCGAAAAACCTCTTCGTCTTCCACTCCAAGACGTCTACAAAATCGGTGGTATCGGTAC

> *glyceralde hyde-3-phosphate dehydrogenase* (*GAPDH*)

AACGCCTCCTGCACCACGAACTGCTTGGCTCCGTTAGCCAAAGTCATCCATGACAAGTTTGGAATCGTTGAAGGTTTGATGACAACTGTCCATGCTACCACCGCAACTCAGAAGACTGTCGATGGGCCTTCTGGAAAGTTATGGCGTGATGGGCGTGGTGCTGCACAAAACATAATTCCAGCATCCACTGGAGCGGCCAAAGCTGTAACTAAAGTGATCAAATCGTTGGAGGGAAAGCTGACCGGCATGGCGTTCCGCGTGCCCG

> *β-actin* (*Actin*)

CGACATGGAAAAGATCTGGCATCACACCTTCTACAACGAACTCCGTGTGGCTCCAGAGGAACACCCCGTACTCTTAACCGAAGCCCCATTGAACCCCAAGGCTAACAGAGAAAAGATGACCCAAATCATGTTCGAAACTTTCAACACCCCAGCCATGTACGTCGCCATCCAAGCCGTCCTTTCCCTGTACGCTTCAGGACGTACCACCGGTATCGTCTTGGACTCTGGTGATGGTGTCTCCCACACCGTACCAATCTACGAAGGTTACGCTCTTCCTCACGCCATCCTCCGTCTTGACTTGGCTGGTCGTGACTTGACCGACTACCTTATGAAAATCCTCACCGAAAGGGGTTACTCATTCACCACCACCGCTGAGAGGGAAATCGTTCGTGACATCAAGGAGAAACTTTGCTATGTCGCCCTCGACTTCGAACAGGAAATGGCCACCGCCGCTGCTTCCACCTCATTGGAGAAATCCTATGAACTTCCCGACGGTCAAGTTATCACCATCGGTAACGAAAGATTCCGTTGCCCTGAAGCCCTCTTCCAACCTTCCTTCTTGGGTATGGAATCCTGCGGTATTCATGAAACTGTCTACAACTCCATCATGAAGTGTGACGTCGATATCCGTAAGGACTTGTACGCCAACACCGTACTCTCTGGTGGTACCACAATGTACCCTGGTATCGCT

> *vacuolar-type H^+^-ATPase subunit A* (*ATPase*)

ACGAGCTGACCCACTCCATATACATCCCTAAAGGTACCAATGTACCTTGCTTATCGAGAACCGCTAAATGGGACTTCAATCCCTGTCATATCAAAATGGGATCTCATTTGACTGGAGGCGACATTTACGGTATCGTCCACGAAAATACGTTGGTTAAACAAAAACTTATGCTACCCCCAAAATCGAAAGGTACAGTAACATACATAGCCGAACCTGGAAGTTATACCGTCGATGATGTCGTTTTGGAAACGGAATTCGACGGTGAACGCACGAAATACACCATGTTGCAAGTTTGGCCCGTGCGTCAACCACGTCCGGTCAGCGAAAAACTACCGGCCAATCATCCTCTGCTCACCGGTCAAAGAGTTCTAGACTCTCTTTTCCCATGTGTTCAAGGTGGTACGACCGCTATACCAGGTGCTTTCGGTTGTGGAAAAACTGTCATTTCGCAGTCGCTGTCCAAATATTCCAACTCTGATGTCATTATTTATGTGGGTTGCGGAGAAAGAGGTAACGAAATGTCCGAGGTACTGCGCGATTTCCCCGAATTGACCGTCGAGATCGAGGGCCAGACGGAATCCATCATGAAACGTACCGCCCTGGTCGCCAACACCTCCAACATGCCCGTGGCCGCCCGCGAGGCCTCCATCTACACCGGCATCACCCTTTCCGAATACTTCAGCGATATGGGCTACAACGTG
